# Search results outliers among MEDLINE platforms

**DOI:** 10.5195/jmla.2019.622

**Published:** 2019-07-01

**Authors:** Christopher Sean Burns, Robert M. Shapiro, Tyler Nix, Jeffrey T. Huber

**Affiliations:** Associate Professor, School of Information Science, University of Kentucky, Lexington, KY, sean.burns@uky.edu; Assistant Professor, School of Information Science, University of Kentucky, Lexington, KY, shapiro.rm@uky.edu; Informationist, Taubman Health Sciences Library, University of Michigan, Ann Arbor, MI, tnix@umich.edu; Professor, School of Information Science, University of Kentucky, Lexington, KY, jeffrey.huber@uky.edu

## Abstract

**Objective:**

Hypothetically, content in MEDLINE records is consistent across multiple platforms. Though platforms have different interfaces and requirements for query syntax, results should be similar when the syntax is controlled for across the platforms. The authors investigated how search result counts varied when searching records among five MEDLINE platforms.

**Methods:**

We created 29 sets of search queries targeting various metadata fields and operators. Within search sets, we adapted 5 distinct, compatible queries to search 5 MEDLINE platforms (PubMed, ProQuest, EBSCO*host*, Web of Science, and Ovid), totaling 145 final queries. The 5 queries were designed to be logically and semantically equivalent and were modified only to match platform syntax requirements. We analyzed the result counts and compared PubMed’s MEDLINE result counts to result counts from the other platforms. We identified outliers by measuring the result count deviations using modified z-scores centered around PubMed’s MEDLINE results.

**Results:**

Web of Science and ProQuest searches were the most likely to deviate from the equivalent PubMed searches. EBSCO*host* and Ovid were less likely to deviate from PubMed searches. Ovid’s results were the most consistent with PubMed’s but appeared to apply an indexing algorithm that resulted in lower retrieval sets among equivalent searches in PubMed. Web of Science exhibited problems with exploding or not exploding Medical Subject Headings (MeSH) terms.

**Conclusion:**

Platform enhancements among interfaces affect record retrieval and challenge the expectation that MEDLINE platforms should, by default, be treated as MEDLINE. Substantial inconsistencies in search result counts, as demonstrated here, should raise concerns about the impact of platform-specific influences on search results.

## INTRODUCTION

The replication and reproduction of research, or lack thereof, is a perennial problem among research communities [1–3]. For systematic reviews and other research that relies on citation or bibliographic records, the evaluation of scientific rigor is partly based on the reproducibility of search strategies. The Preferred Reporting Items for Systematic Reviews and Meta-Analyses (PRISMA) Guidelines and the Cochrane Handbook for Systematic Reviews and Interventions are examples of how scholars recognize the need for systematic reporting of methods and the organization of review research [4, 5].

Differences in search interfaces, article indexing, and retrieval algorithms also impact reproducibility and replication, which are important aspects of the scientific process, evidence-based medicine, and the creation of systematic reviews [6–12]. Even if search strategies are methodical and well documented, searches might not be reproducible because many platforms are proprietary products, and thus the code, algorithms, and, in general, the software that drives these products are not available for public review. Consequently, one can only speculate how such systems work by inference from use; for example, by comparing them to similar products [13, 14].

Although the National Library of Medicine (NLM) maintains the MEDLINE records and provides free (i.e., federally subsidized) access to them through PubMed, they also license these records to database vendors (hereafter, “platforms”). Furthermore, although these platforms operate with the same MEDLINE data, each platform applies its own indexing technologies and its own search interface, and it is possible that these alterations influence different search behaviors and retrieval sets [15, 16].

Some studies used queries that were designed to study reproducibility across platforms by comparing recall and precision for retrieval sets across platforms [17–19]. However, different query syntax across platforms has been highlighted as an important problem itself [20, 21]. One small study, for example, compared search queries and results among different interfaces to the CINAHL database and reported reproducible search strategies except for queries that contained subject-keyword terms [22]. Another study reported that different interfaces to the same underlying database or set of records produced different search results and noted that the practical implications of missing a single record from a literature review could skew results or alter the focus of a study [23]. A third study found that PubMed retrieved more records than Ovid’s MEDLINE, but this study did not include MEDLINE subset results in PubMed [24]. A reply to this study suggested that the differences could be explained by basic problems with bibliographic and MEDLINE searching and concluded that “database and search interface providers should agree on common standards in terminology and search semantics and soon make their professional tools as useful as they are intended to” [25].

The purpose of this study was to document how different MEDLINE platforms influenced search result counts (presumably based on the same MEDLINE data file) by creating equivalent, structured, and straightforward queries to search across these platforms (i.e., by controlling for query syntax). The authors asked the research question: how much do search result counts among MEDLINE platforms vary after controlling for search query syntax?

## METHODS

We examined five MEDLINE platforms by creating twenty-nine sets of search queries for each platform and comparing search count results. The platforms were PubMed’s MEDLINE subset, ProQuest’s MEDLINE, EBSCO*host*’s MEDLINE, Web of Science’s MEDLINE, and Ovid’s MEDLINE, hereafter simply referred to by their main platform name (e.g., PubMed, Ovid).

Our queries were organized into 29 sets, with each set containing 5 equivalent queries, 1 per platform, and numbered sequentially (s01, s02…s29), for a total of 145 searches. Two authors collected the counts for all platforms by running the queries in the platforms and recording the total records returned in a spreadsheet. PubMed search counts were recorded on results sorted by *most recent* since PubMed alters the search query, and thus the search results, when sorting by *best match* [26]. The other MEDLINE platforms do not alter search records or counts based on sorting parameters.

Each of the 29 search sets targeted various search operators and metadata fields. For example, Table 1 reports an example set of queries and search result counts for search set s09 (composed of a single MeSH term appearing on a single branch of the MeSH tree, exploded, and combined with a keyword and date limit). All 145 queries, search logic descriptions, and search count results are provided in supplemental Appendix A. Some of our queries were limited by publication dates so that we could limit the influence of records that have been newly added and reduce deviations based on updates to PubMed and then updates to the other platforms.

**Table 1:** Example search queries and results for search set s09

	Search set s09	Result counts
PubMed	“neoplasms”[MH] AND “immune”[ALL] AND 1950:2015[DP]	72,297
ProQuest	MESH.EXPLODE(“neoplasms”) AND NOFT(“immune”) AND YR(1950–2015)	72,641
EBSCO*host*	MH(“neoplasms+”) AND TX(“immune”) AND YR 1950–2015	72,987
Web of Science	MH:exp=(“neoplasms”) AND TS=(“immune”) AND PY=(1950–2015)	14,711
Ovid	1. EXP neoplasms/ AND immune.AF 2. limit 1 to YR=1950–2015	71,594

Table 1 represents how the five queries per set were designed to be semantically and logically equivalent and were modified only to match the syntax required by each platform. In another example, our first set of queries (s01) compared the same all-field keyword search (e.g., “neoplasms”[All] AND medline[SB] in PubMed) across these five platforms, and our second set of queries (s02) compared the same single MeSH term (single branch, no explode) searches across platforms (supplemental Appendix A). The remaining queries were constructed to explore other permutations of simple searches, including searches with single MeSH terms on single and multiple branches as well as other field searches like journal titles, author names, and date limits. Queries were constructed to specifically search the MEDLINE subset of each platform when it was not the default. For example, PubMed queries that did not contain MeSH terms included the limiter “medline[sb]”, and all Ovid queries were run in the “mesz” segment, which includes only documents with MEDLINE status and omits epub ahead of print, in-process, and other non-indexed records contained in the “ppez” segment.

The queries were not designed to mimic end user usage nor were they designed to examine database coverage. Rather, they were designed to explore search result counts stemming from basic query syntax and differences in search field indexing. That is, our goal was to understand baseline deviations and to detect outliers to help understand whether reproducing queries across MEDLINE platforms is hindered by the platforms. All searches were created and pilot-tested in the summer of 2018. The results reported here are from searches conducted in October 2018.

To answer our research question, our analysis is based on a comparison of search result counts and modified z-scores (*m_i_*) for the result counts in each search set. The modified z-score is a version of the standard z-score and is likewise interpreted and applicable in locating deviations; however, it is more robust against outliers [27]. Generally, the standard z-score is compared to the mean (or the center of the data), but we centered our scores around the PubMed result counts from each search set to highlight search result counts that deviate from those of PubMed. In particular, we defined search result outliers as any modified z-score that deviated more than ±3.5 from PubMed, as recommended by Iglewicz and Hoaglin [27]. In addition to the z-score, we highlighted search count differentials (result counts as compared to those of PubMed) for all searches, as reported in the table in supplemental Appendix B. Even if results do not deviate from PubMed by ±3.5 standardized points, differences in counts help highlight deviations across MEDLINE platforms.

The analysis was conducted in the R programming language with additional software libraries [28–34]. Code and data for this analysis are provided in supplemental Appendixes C and D.

## RESULTS

Overall, we found that most searches resulted in retrieval differences among MEDLINE platforms and that some platforms deviated from PubMed more than others. In general, ProQuest and EBSCO*host* exhibited similar patterns of search result count deviations from PubMed, but ProQuest deviated from PubMed more substantially, with three search queries classified as outliers. Web of Science exhibited the most idiosyncratic search result count deviations from PubMed searches, with five search queries returning substantially different counts. Although Ovid’s search result counts showed fewer and less exaggerated deviations, it consistently returned fewer records than PubMed, even for publications restricted by publication date range 1950–2015. This deviation suggested that there was an important difference between PubMed and Ovid in how they indexed their records. By fixing the publication dates to a range, ongoing updates to the database content should have had less influence on these differences.

**Figure 1:**
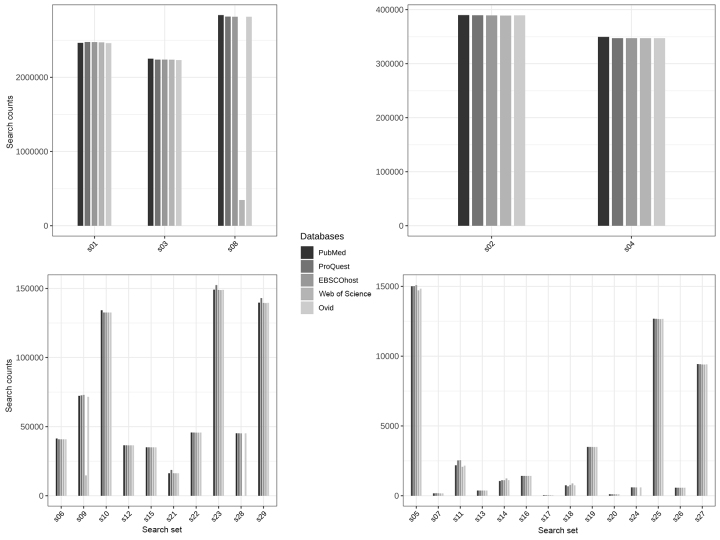
Total search result counts for each of the 29 search sets The four plots are organized by the magnitude of results.

Figure 1 shows the total records returned for each set of queries. The figure is faceted into 4 plots by the magnitude of search result counts. On the surface, most searches in each set appear to be consistent with the others. However, there are a few obvious inconsistencies in the results. For example, the Web of Science search returned only 20% of the search records that PubMed returned for the equivalent query in search set s09 (Table 1) and only 12% of the search records that PubMed returned in search set s08 (Table 1). In both searches, the queries exploded the MeSH term “Neoplasms,” indicating a problem with how Web of Science explodes terms.

To derive the search result differentials, we subtracted each query’s total number of search count results from the PubMed search count results in the respective set to analyze how far each search deviated from PubMed. We also examined the search differentials using a modified z-score, which allowed us to zoom in on the discrepancies. (The table in supplemental Appendix B reports the differentials.) For example, in search set s10, PubMed returned 134,217 records with publication dates limited from 1950–2015 for a search against the MeSH term “Dementia,” exploded. The other 4 platforms returned between 1,618 to 1,627 fewer records.

These deviations in this set were fairly consistent across the 4 platforms and statistically small, per the z-scores (indicated in parentheses in supplemental Appendix B). However, search set s23 also queried for “Dementia” (exploded) but did not limit results by publication date. Here, PubMed returned 149,146 total records, and the other 4 platforms returned a more varied number of results. In this case, ProQuest returned the greatest differential and was a statistical outlier, by retrieving 3,266 more records than the equivalent PubMed search. The remaining 3 platforms returned fewer records than the PubMed search, although they were closer, ranging from 167 to 348 fewer records.

**Figure 2:**
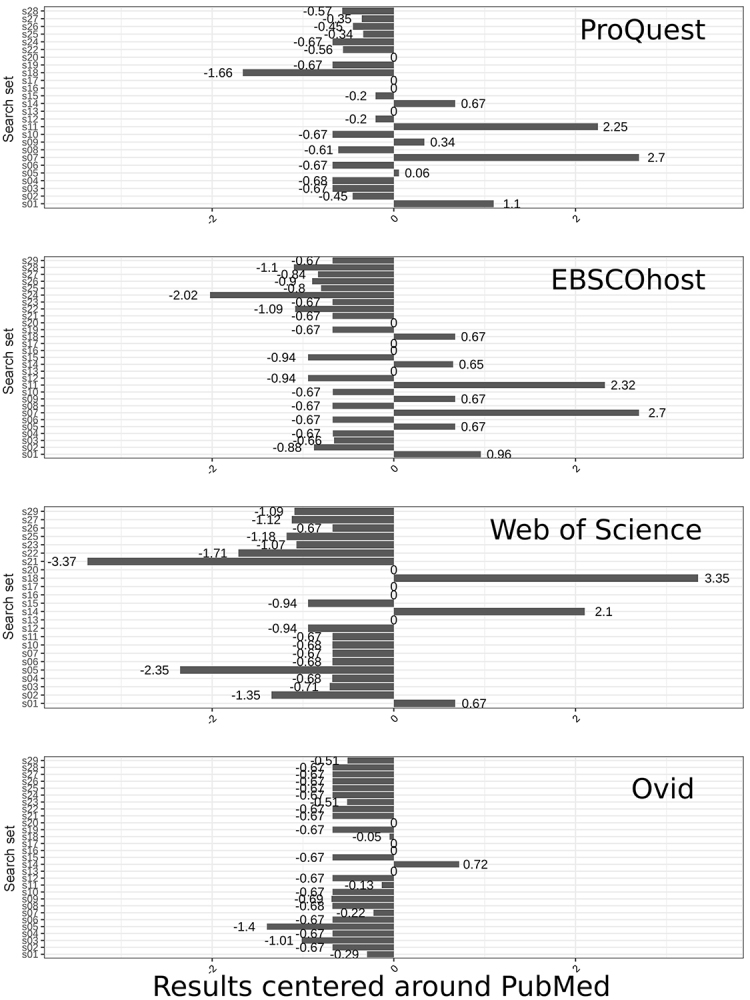
Deviations per platform from PubMed’s MEDLINE, excluding outlier searches

**Figure 3:**
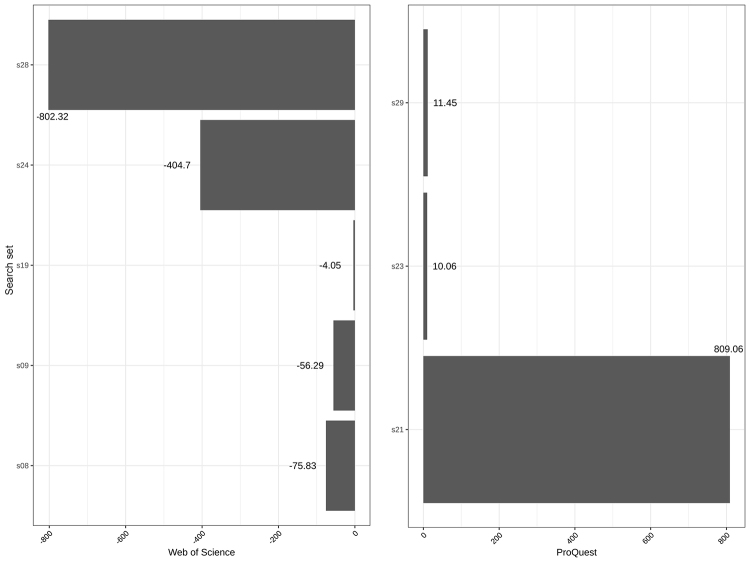
Outlier search results in ProQuest and Web of Science Numbers represent modified z-scores. A score outside of +/−3.5 is considered an outlier.

Figures 2 and 3 present the z-scores and highlight the deviations for all searches compared to PubMed. Figure 2 includes the search sets within ±3.5 deviations from PubMed, and Figure 3 includes deviations outside that range that are, therefore, classified as outliers. In both figures, PubMed results are represented by the center, that is, 0 deviations.

Figure 2 highlights the substantial inconsistencies between PubMed and the 4 platforms and low consistency in the deviation across the 4 platforms themselves. For example, search s07 (a single MeSH term, “Dementia,” not exploded, with an additional keyword, “immune,” and a date restriction) shows that ProQuest and EBSCO*host* returned results equivalent to an average of 2.7 more records than PubMed (Figure 2; supplemental Appendix B). However, that same search returned fewer average records for Ovid and Web of Science. Two searches (s16 and s17) consistently retrieved the same number of results across all platforms. A third search (s13) retrieved the same results across PubMed, ProQuest, EBSCO*host*, and Ovid but not Web of Science, and a fourth search (s20) was consistent across PubMed, ProQuest, EBSCO*host*, and Web of Science but not Ovid. In searches s13 and s20, respectively, Web of Science was only 2 results below the other platforms, and Ovid retrieved only 1 fewer result.

Figure 3 shows the outliers, defined as search result counts beyond ±3.5 standard deviations away from PubMed. Only Web of Science and ProQuest had search result count outliers, with each for different search sets. All Web of Science outliers included results that returned fewer records than PubMed, and all ProQuest outliers included results that returned more records than PubMed.

Two of the high outliers for ProQuest searches included at least 1 MeSH term that appeared on multiple branches and that were exploded (s23, *m_i_*=10.06; s29, *m_i_*=11.45) (Figure 3). In both searches, ProQuest returned thousands more results than PubMed. Web of Science (s23, *m_i_*=–1.07; s29, *m_i_*=−1.09) also deviated by more than 1 standard deviation from PubMed in these 2 searches, but in the opposite direction, returning fewer records. EBSCO*host* (s23, *m_i_*=−0.67; s29, *m_i_*=−0.67) and Ovid (s23, *m_i_*=−0.51; s29, *m_i_*=−0.51) also returned fewer results, but these results were much closer to PubMed’s. Similar differences are seen in search s21, in which ProQuest retrieved thousands more results than PubMed (*m_i_*=809.06), although the other 3 platforms retrieved fewer results than the PubMed baseline. This search examined the equivalent of “All Fields” across the platforms combined with 2 journal titles.

As stated, Web of Science result counts deviated most often from PubMed searches. In the 15 Web of Science searches that deviated from the equivalent PubMed searches by at least 1 standard deviation, 5 of those searches were extreme outliers (s08, s09, s19, s24, and s28; supplemental Appendix A). The first 2 searches (s08, *m_i_*=−75.83; s09, *m_i_*=−56.29) highlighted issues with how Web of Science exploded MeSH terms. The third search (s19, *m_i_*=−4.05) returned only 6 fewer records than PubMed but is considered an outlier relative to how closely the other 3 platforms matched PubMed’s results. The fourth search (s24, *m_i_*=−404.07) returned 0 records even though PubMed retrieved 600 records, and the other 3 platforms returned approximately the same. The fifth search (s28, *m_i_*=−802.32) returned 0 records, compared to over 45,000 records retrieved on the other 4 platforms, with a query that included 2 MeSH terms.

Author name searches were problematic across the platforms except when they were attached to MeSH terms, which seemed to help disambiguate the names (s17 and s18; supplemental Appendix A). In a search for a single author name only, Ovid (s18, *m_i_*=−0.05) returned results that were nearly equal with PubMed results. However, by increasing magnitude, EBSCO*host* (*m_i_*=0.67) returned more results, ProQuest (*m_i_*=−1.66) returned fewer results, and Web of Science (*m_i_*=3.35) returned more than PubMed. When the author name was attached to 2 MeSH terms (s17; supplemental Appendix A), all 4 platforms returned the same number of results as PubMed.

We found that very specific search queries were more likely to produce more consistent results across all five platforms. In addition to the search query described above that included MeSH terms and a single author name (s17; supplemental Appendix A), there were searches that resulted in perfect or nearly perfect agreement among all platforms (s13, s16, and s20; supplemental Appendix A). The first of these searches (s13) included two MeSH terms and a title keyword and exploded the second MeSH term. The second of these searches (s16) included two MeSH terms (one not exploded and one exploded) joined by a Boolean NOT and searched against one journal title. The third of these searches (s20) included a title term search against two journal titles.

Likewise, four other searches produced fewer records than PubMed but near consistent results among each other (s4, s6, s10, and s26). These were also very specific searches, including only MeSH terms. In addition, the first three of these searches were limited by publication dates. However, including only specific terms did not guarantee consistent results across all platforms. In particular, Web of Science often deviated from the others when only MeSH terms were included in the query. The deviations were likely the result of how Web of Science explodes terms.

## DISCUSSION

In this research, we constructed queries across five MEDLINE platforms in order to understand how search result counts vary after controlling for necessary differences in search query syntax across platforms. Hypothetically, content in the MEDLINE platforms is consistent across platforms because each uses MEDLINE records created by NLM. However, this assumption has lacked thorough scientific testing, which can be problematic, especially if studies combine multiple MEDLINE platforms under a single “MEDLINE” category [35]. Although one might expect some variation in search results across platforms since search interfaces and syntax are vendor-specific; in general, search results should be similar, if not identical, for queries that are equivalent.

It appears, however, that no MEDLINE platform can be a substitute for another MEDLINE platform, which is problematic if researchers, clinicians, and health information professionals do not have access to all of them and, thus, do not have the ability to cross-reference searches and de-duplicate search records when they conduct literature searches. The inability to substitute one MEDLINE platform for another can be caused by various interventions by platform vendors (possibly including data ingest workflows, term indexing and retrieval algorithms, and interface features) that affect record retrieval. Hence, our results challenge the expectation that all MEDLINE platforms produce equivalent results and that they should be treated as MEDLINE. The inconsistencies seen here across platforms should raise concerns about the impact of vendor-specific indexing algorithms. It appears that the features provided by the proprietary platforms have a significant impact on the retrieved results of even basic queries. This, in turn, affects the replication and reproducibility of search query development and, possibly, the conclusions drawn from those literature sets.

Practically speaking, the queries that returned the most similar result counts to their equivalent PubMed searches were multifaceted and included either MeSH terms or a title keyword and then were combined with another field, such as a journal title, author name, or journal search (e.g., s13, s16, s17, s20). However, deviations were not generally consistent across platforms nor in relation to specific query elements (e.g., specific metadata combinations). As such, there appear to be no ready solutions for mitigating inconsistencies in search results across platforms as an end user. Because perhaps few users have access to all MEDLINE platforms, this could be problematic, since, as noted, even one missing study can skew or alter scientific or clinical conclusions [23].

Although Ovid produced the most consistent results with PubMed, there were still differences in search result counts. In all those cases where Ovid and PubMed differed, Ovid returned fewer results (without de-duplicating). We were able to rule out that these differences were solely the result in lag time between MEDLINE updates, that is, the time between when PubMed is updated and the licensed platforms are updated, because the Ovid search counts were lower even for those queries that were limited by publication dates (1950–2015).

Without knowing what has been left out of these search results, it would be difficult to know how those results might impact clinical care, especially because MEDLINE has been deemed an important source for practice and where even one record can have important consequences in treatment [35, 36]. As such, future studies should include research questions related to understanding the contents of retrieved sets in order to understand how the bibliographic records are influencing retrieval across the platforms.

Lag time between updates of the MEDLINE file across the platforms also could not explain differences in results for ProQuest and EBSCO*host*. The higher counts in ProQuest and in EBSCO*host* suggested that their indexing algorithms were more sensitive and defaulted to more inclusive retrieval sets. This claim was supported by ProQuest’s highest outlier, which included a keyword search against 2 specific journal titles (s21, *m_i_*=179.42; supplemental Appendix A). Given the variances observed across platforms, it is important to understand under what conditions queries across MEDLINE platforms might be more sensitive.

One limitation of our study is that it is only a snapshot at one moment. Therefore, future studies could examine longitudinal changes in how these systems respond to basic searches to increase understanding of the effects that vendor-specific algorithms have on search result counts, because it could be that such algorithms are modified over time. Additional lines of research include examining how retrieval of non-indexed and in-process citations in PubMed’s MEDLINE subset differentiate from comparable databases or subsets.

Also, as noted earlier, this study examined baseline differentiation for permutations of simple searches. However, searches documented in the literature for, among other things, systematic reviews should also be compared across platforms. Such studies could help researchers understand the maximum differentiation that these systems might exhibit since the queries documented in these studies are generally complex.

We also used PubMed search counts as the point of reference, and we did this because NLM is responsible for both MEDLINE and the PubMed interface. However, other platforms could function as a point of reference and doing so might be useful in explaining differences in indexing, Boolean logic, and other aspects of searching. Lastly, although analyzing baseline deviation using search counts helps illustrate fundamental differences among MEDLINE platforms, future research could examine and compare the content of records that are returned to better understand the source of these deviations.

## SUPPLEMENTAL FILES

Appendix A All queries, search logic descriptions, and search count results

Appendix B Search differentials (search count and modified z-score differences) with PubMed as the reference point for each search set

Appendix D R programming code used to analyze the data

Appendix D Data set formatted for use in the R programming language
