# Supplementary material for: Search results outliers among MEDLINE platforms
Source: J Med Libr Assoc. 2019 Jul 1;107(3):364–73. doi: 10.5195/jmla.2019.622 (PMC6579582; doi:10.5195/jmla.2019.622)
Supplement: Appendix B [file jmla-107-364-s002.pdf]

Journal of the Medical Library Association

Supplemental content to
J Med Libr Assoc. Jul;107(3):dx.doi.org/10.5195/jmla.2019.622
www.jmla.mlanet.org
© Burns, Shapiro II, Nix, Huber 2019

Search results outliers among MEDLINE platforms

Christopher Sean Burns; Robert M. Shapiro II; Tyler Nix; Jeffrey T. Huber

APPENDIX B

Search differentials (search count and modified z-score differences) with PubMed as the reference point for each search set

Search sets	PubMed (reference point)	ProQuest		EBSCOhost		Web of Science		Ovid	
		search count	(z-score)	search count	(z-score)	search count	(z-score)	search count	(z-score)
s01	2,463,875	11,849	(1.10)	10,331	(0.96)	7,277	(0.68)	−3,180	(−0.29)
s02	389,955	−281	(−0.45)	−545	(−0.88)	−835	(−1.35)	−418	(−0.67)
s03	2,251,033	−12,335	(−0.67)	−12,036	(−0.66)	−12,915	(−0.71)	−18,553	(−1.01)
s04	349,598	−2,406	(−0.68)	−2,393	(−0.67)	−2,415	(−0.68)	−2,403	(−0.67)
s05	15,004	7	(0.06)	84	(0.67)	−293	(−2.35)	−174	(−1.40)
s06	41,444	−583	(−0.67)	−582	(−0.67)	−584	(−0.68)	−583	(−0.67)
s07	172	12	(2.70)	12	(2.70)	−3	(−0.67)	−1	(−0.23)
s08	2,838,188	−20,095	(−0.61)	−22,158	(−0.67)	−2,491,005	(−75.83)	−22,213	(−0.68)
s09	72,297	344	(0.34)	690	(0.67)	−57,586	(−56.29)	−703	(−0.69)
s10	134,217	−1,624	(−0.67)	−1,618	(−0.67)	−1,627	(−0.68)	−1,624	(−0.67)
s11	2,181	353	(2.25)	365	(2.32)	−106	(−0.67)	−21	(−0.13)
s12	36,503	−6	(−0.20)	−28	(−0.94)	−28	(−0.94)	−20	(−0.67)
s13	377	0	(0.00)	0	(0.00)	−2	(0.00)	0	(0.00)
s14	1,054	61	(0.67)	59	(0.65)	190	(2.10)	65	(0.72)
s15	35,080	−6	(−0.20)	−28	(−0.94)	−28	(−0.94)	−20	(−0.67)
s16	1,423	0	(0.00)	0	(0.00)	0	(0.00)	0	(0.00)
s17	41	0	(0.00)	0	(0.00)	0	(0.00)	0	(0.00)
s18	747	−69	(−1.66)	28	(0.67)	139	(3.35)	−2	(−0.05)
s19	3,495	−1	(−0.67)	−1	(−0.67)	−6	(−4.05)	−1	(−0.67)
s20	111	0	(0.00)	0	(0.00)	0	(0.00)	−1	(0.00)
s21	16,291	2,399	(809.06)	−2	(−0.67)	−10	(−3.37)	−2	(−0.67)
s22	45,782	−34	(−0.56)	−66	(−1.09)	−104	(−1.71)	−41	(−0.67)
s23	149,146	3,266	(10.06)	−219	(−0.67)	−348	(−1.07)	−167	(−0.51)
s24	600	−1	(−0.67)	−3	(−2.02)	−600	(−404.70)	−1	(−0.67)
s25	12,680	−8	(−0.34)	−19	(−0.80)	−28	(−1.18)	−16	(−0.67)
s26	581	−2	(−0.45)	−4	(−0.90)	−3	(−0.67)	−3	(−0.67)
s27	9,432	−11	(−0.35)	−26	(−0.83)	−35	(−1.12)	−21	(−0.67)
s28	45,201	−32	(−0.57)	−62	(−1.10)	−45,201	(−802.32)	−38	(−0.67)
s29	139,714	3,277	(11.45)	−193	(−0.67)	−313	(−1.09)	−146	(−0.51)
