# Supplementary material for: Search results outliers among MEDLINE platforms
Source: J Med Libr Assoc. 2019 Jul 1;107(3):364–73. doi: 10.5195/jmla.2019.622 (PMC6579582; doi:10.5195/jmla.2019.622)
Supplement: Appendix D [file jmla-107-364-s003.pdf]

Journal of the Medical Library Association

Supplemental content to
J Med Libr Assoc. Jul;107(3):dx.doi.org/10.5195/jmla.2019.622
www.jmla.mlanet.org
© Burns, Shapiro II, Nix, Huber 2019

Search results outliers among MEDLINE platforms

Christopher Sean Burns; Robert M. Shapiro II; Tyler Nix; Jeffrey T. Huber

APPENDIX C

R programming code used to analyze the data

```
# Source packages
# gridExtra is for a multiplot
library("magicfor")
library("ggplot2")
library("gridExtra")
library("dplyr")
library("reshape2")
library("xtable")

# Read search counts
source("data.R")

# Combine search counts
searches <- rbind(s01, s02, s03, s04, s05, s06, s07, s08, s09, s10, s11, s12,
                  s13, s14, s15, s16, s17, s18, s19, s20, s21, s22, s23, s24,
                  s25, s26, s27, s28, s29)
searches <- as.data.frame(searches)

# Name columns/variables
colnames(searches) <- c("pubmed", "proquest", "ebscohost", "wos", "ovid")

# Create column for search sets
searches$searchset <- rownames(searches)

# Cleanup
rm(s01, s02, s03, s04, s05, s06, s07, s08, s09, s10, s11, s12, s13, s14, s15,
   s16, s17, s18, s19, s20, s21, s22, s23, s24, s25, s26, s27, s28, s29)

# Calculate modified z-score $m_i$
# function based on pubmed count and not median as center
modz <- function(searches, x) {
  s    <- as.numeric(searches[x, 1:5])
  sabs  <- abs(s - s[1])
```

JMLA Journal of the Medical Library Association
SC
Supplemental content to
J Med Libr Assoc. Jul;107(3):dx.doi.org/10.5195/jmla.2019.622
www.jmla.mlanet.org
© Burns, Shapiro II, Nix, Huber 2019

```
  smad  <- median(sabs)
  smodz <- (0.6745 * (s - s[1])) / smad
  return(smodz)
}

# save output of for loop into a dataframe; bless this package!
magic_for(print, silent = TRUE)

for (i in 1:29) {
  print(modz(searches, i))
}

modz <- magic_result_as_vector()
modz <- as.data.frame(matrix(modz, ncol = 5, byrow = T))

# Name columns/variables
colnames(modz) <- c("pubmed", "proquest", "ebscohost", "wos", "ovid")

# Replace NaNs and Infs with 0 to show perfect correlation since they are
# perfectly or nearly perfectly correlated
modz[c(13, 16, 17, 20), ] <- c(0, 0, 0, 0)

# Create column for search sets
modz$searchset <- rownames(searches)

##### FIGURE 1: Raw Scores #####
# To convert 'searches' data frame in long format for grouped bar chart
searcheslong <- melt(searches, id = "searchset")
colnames(searcheslong) <- c("searchset", "databases", "searchcount")

# `options` will avoid scientific notation
options(scipen = 10000)

# 1a: Separate high counts to help with scaling
fighighcounts <- filter(searcheslong,
                        searchset == "s01" |
                        searchset == "s03" |
                        searchset == "s08")
```

Supplemental content to
J Med Libr Assoc. Jul;107(3):dx.doi.org/10.5195/jmla.2019.622
www.jmla.mlanet.org
© Burns, Shapiro II, Nix, Huber 2019

```
fig1hc <- ggplot(fighighcounts, aes(searchset, searchcount)) +
  geom_bar(aes(fill = databases), width = 0.4,
           position = position_dodge(width = 0.5),
           stat = "identity") + labs(x = "", y = "Search Counts") +
theme_bw() + theme(axis.text.x = element_text(angle = 45, hjust = 1,
                                 colour = "black")) +
theme(axis.text.y = element_text(size = 10)) +
scale_fill_grey(name = "Databases",
             breaks = c("pubmed", "proquest", "ebscohost",
                   "wos", "ovid"),
             labels = c("PubMed", "ProQuest", "EBSCOhost",
                   "WoS", "Ovid"))

# 1b: Separate high moderate counts to help with scaling
figmodcounts <- filter(searcheslong,
                  searchset == "s02" |
                  searchset == "s04")

fig1mc <- ggplot(figmodcounts, aes(searchset, searchcount)) +
  geom_bar(aes(fill = databases), width = 0.4,
           position = position_dodge(width = 0.5),
           stat = "identity") + labs(x = "", y = "") +
theme_bw() + theme(axis.text.x = element_text(angle = 45, hjust = 1,
                                 colour = "black")) +
theme(axis.text.y = element_text(size = 10)) +
scale_fill_grey(guide = FALSE, name = "Databases",
             breaks = c("pubmed", "proquest", "ebscohost",
                   "wos", "ovid"),
             labels = c("PubMed", "ProQuest", "EBSCOhost",
                   "WoS", "Ovid"))

# 1c: Separate low moderate counts to help with scaling
figmidcounts <- filter(searcheslong,
                 searchset == "s06" |
                 searchset == "s09" |
                 searchset == "s10" |
                 searchset == "s12" |
                 searchset == "s15" |
                 searchset == "s21" |
```

JMLA Journal of the Medical Library Association

SC
Supplemental content to
J Med Libr Assoc. Jul;107(3):dx.doi.org/10.5195/jmla.2019.622
www.jmla.mlanet.org
© Burns, Shapiro II, Nix, Huber 2019

```
            searchset == "s22" |
            searchset == "s23" |
            searchset == "s28" |
            searchset == "s29")

fig1mic <- ggplot(figmidcounts, aes(searchset, searchcount)) +
  geom_bar(aes(fill = databases), width = 0.4,
        position = position_dodge(width = 0.5),
        stat = "identity") + labs(x = "Search Set", y = "Search Counts") +
theme_bw() + theme(axis.text.x = element_text(angle = 45, hjust = 1,
                         colour = "black")) +
theme(axis.text.y = element_text(size = 10)) +
scale_fill_grey(name = "Databases",
          breaks = c("pubmed", "proquest", "ebscohost",
               "wos", "ovid"),
          labels = c("PubMed", "ProQuest", "EBSCOhost",
               "WoS", "Ovid"))

# 1d: Separate low counts to help with scaling
figlowcounts <- filter(searcheslong,
            searchset != "s01" &
            searchset != "s03" &
            searchset != "s02" &
            searchset != "s04" &
            searchset != "s06" &
            searchset != "s08" &
            searchset != "s09" &
            searchset != "s10" &
            searchset != "s12" &
            searchset != "s15" &
            searchset != "s21" &
            searchset != "s22" &
            searchset != "s23" &
            searchset != "s28" &
            searchset != "s29")

fig1lc <- ggplot(figlowcounts, aes(searchset, searchcount)) +
  geom_bar(aes(fill = databases), width = 0.4,
        position = position_dodge(width = 0.5),
```

```
        stat = "identity") + labs(x = "Search Set", y = "") +
theme_bw() + theme(axis.text.x = element_text(angle = 45, hjust = 1,
                                  colour = "black")) +
theme(axis.text.y = element_text(size = 10)) +
scale_fill_grey(guide = FALSE, name = "Databases",
          breaks = c("pubmed", "proquest", "ebscohost",
                "wos", "ovid"),
          labels = c("PubMed", "ProQuest", "EBSCOhost",
                "WoS", "Ovid"))

fig1 <- grid.arrange(fig1hc, fig1mc, fig1mic, fig1lc, ncol = 2, nrow = 2)

ggsave("plots/figure-1-raw.svg", plot = fig1, height = 9,
    width = 12, dpi = 300)

ggsave("plots/figure-1-raw.png", plot = fig1, device = png(), height = 9,
    width = 12, dpi = 300)

dev.off()

##### FIGURE 2: Raw Score Diffs from PubMed MEDLINE #####
raw1.diff <- ggplot(searches, aes(searchset, proquest - pubmed)) +
  geom_col() +
  labs(x = "Search Set", y = "ProQuest") + theme_bw() + coord_flip() +
    theme(axis.text.x = element_text(angle = 45,
                        hjust = 1,
                        colour = "black"))

raw2.diff <- ggplot(searches, aes(searchset, ebscohost - pubmed)) +
  geom_col() +
  labs(x = "", y = "EBSCOhost") + theme_bw() + coord_flip() +
    theme(axis.text.x = element_text(angle = 45,
                        hjust = 1,
                        colour = "black"))

raw3.diff <- ggplot(searches, aes(searchset, wos - pubmed)) +
  geom_col() +
  labs(x = "Search Set", y = "Web of Science") + theme_bw() + coord_flip() +
    theme(axis.text.x = element_text(angle = 45,
```

Supplemental content to
J Med Libr Assoc. Jul;107(3):dx.doi.org/10.5195/jmla.2019.622
www.jmla.mlanet.org
© Burns, Shapiro II, Nix, Huber 2019

```
                           hjust = 1,
                           colour = "black"))

raw4.diff <- ggplot(searches, aes(searchset, ovid - pubmed)) +
  geom_col() +
  labs(x = "", y = "Ovid") + theme_bw() + coord_flip() +
      theme(axis.text.x = element_text(angle = 45,
                           hjust = 1,
                           colour = "black"))

fig2 <- grid.arrange(raw1.diff, raw2.diff, raw3.diff, raw4.diff,
                ncol = 2, nrow = 2)

ggsave("plots/figure-2-raw-diffs.svg", plot = fig2, height = 9, width = 12,
      dpi = 300)

ggsave("plots/figure-2-raw-diffs.png", device = png(), plot = fig2, height = 9,
      width = 12, dpi = 300)

dev.off()

##### FIGURE 3: Modified z scores #####
# Remove extreme outliers
modz1 <- subset(modz, proquest <= 3.5 & proquest >= -3.5)
mz1 <- ggplot(modz1, aes(searchset, proquest, label = round(proquest, 2))) +
  geom_col() + geom_text() +
  labs(x = "Search Set", y = "") + theme_bw() +
  coord_flip(ylim = c(-3.5, 3.5), xlim = c(1, 26)) +
  annotate("text", x = 20, y = 2, label = "ProQuest") +
      theme(axis.text.x = element_text(angle = 45,
                           hjust = 1,
                           colour = "black"))

modz2 <- subset(modz, ebscohost <= 3.5 & ebscohost >= -3.5)
mz2 <- ggplot(modz2, aes(searchset, ebscohost, label = round(ebscohost, 2))) +
  geom_col() + geom_text() +
  labs(x = "Search Set", y = "") + theme_bw() +
  coord_flip(ylim = c(-3.5, 3.5), xlim = c(1, 29)) +
  annotate("text", x = 20, y = 2, label = "EBSCOhost") +
```

JMLA Journal of the Medical Library Association
Supplemental content to
J Med Libr Assoc. Jul;107(3):dx.doi.org/10.5195/jmla.2019.622
www.jmla.mlanet.org
© Burns, Shapiro II, Nix, Huber 2019

```
    theme(axis.text.x = element_text(angle = 45,
                                     hjust = 1,
                                     colour = "black"))

modz3 <- subset(modz, wos <= 3.5 & wos >= -3.5)
mz3 <- ggplot(modz3, aes(searchset, wos, label = round(wos, 2))) +
  geom_col() + geom_text() +
  labs(x = "Search Set", y = "") + theme_bw() +
  coord_flip(ylim = c(-3.5, 3.5), xlim = c(1, 24)) +
  annotate("text", x = 20, y = 2, label = "Web of Science") +
    theme(axis.text.x = element_text(angle = 45,
                                     hjust = 1,
                                     colour = "black"))

modz4 <- subset(modz, ovid <= 3.5 & ovid >= -3.5)
mz4 <- ggplot(modz4, aes(searchset, ovid, label = round(ovid, 2))) +
  geom_col() + geom_text() +
  labs(x = "Search Set", y = "") + theme_bw() +
  coord_flip(ylim = c(-3.5, 3.5), xlim = c(1, 29)) +
  annotate("text", x = 20, y = 2, label = "Ovid") +
    theme(axis.text.x = element_text(angle = 45,
                                     hjust = 1,
                                     colour = "black"))

fig3 <- grid.arrange(mz1, mz2, mz3, mz4, ncol = 1, nrow = 4)

ggsave("plots/figure-3-modz-scores.svg", plot = fig3,
    height = 12, width = 9, dpi = 300)

ggsave("plots/figure-3-modz-scores.png", plot = fig3, device = png(),
    height = 12, width = 9, dpi = 300)

dev.off()

##### FIGURE 4: ProQuest, Web of Science Extreme Outliers #####
# Plot WoS extreme outliers
modzwosoutliers <- subset(modz, wos >= 3.5 | wos <= -3.5)
modzwosout <- ggplot(modzwosoutliers, aes(searchset, wos,
                                          label = round(wos, 2))) +
```

© Burns, Shapiro II, Nix, Huber 2019

```
  geom_col() + geom_text() +
  labs(x = "Search Set", y = "Web of Science") + theme_bw() + coord_flip() +
      theme(axis.text.x =
            element_text(angle = 45, hjust = 1, colour = "black"))

# Plot ProQuest extreme outliers
modzprouqestoutliers <- subset(modz, proquest >= 3.5 | proquest <= -3.5)
modzpqout <- ggplot(modzprouqestoutliers, aes(searchset, proquest,
                                label = round(proquest, 2))) +
  geom_col() + geom_text() +
  labs(x = "", y = "ProQuest") + theme_bw() + coord_flip() +
  theme(axis.text.x = element_text(angle = 45, hjust = 1, colour = "black"))

fig4 <- grid.arrange(modzwosout, modzpqout, ncol = 2, nrow = 1)

ggsave("plots/figure-4-modz-ext-outliers.svg", plot = fig4,
      height = 9, width = 12, dpi = 300)

ggsave("plots/figure-4-modz-ext-outliers.png", plot = fig4, device = png(),
      height = 9, width = 12, dpi = 300)

dev.off()

# Save data as tables tables and incorporate into LibreOffice
modztable <- as.data.frame(round(modz[, c(2, 3, 4, 5)], 3))

searchdiffs <- as.data.frame(cbind(searches$proquest - searches$pubmed,
                         searches$ebscohost - searches$pubmed,
                         searches$wos - searches$pubmed,
                         searches$ovid - searches$pubmed))

colnames(searchdiffs) <- c("proquest", "ebscohost", "wos", "Ovid")

searchdiffs$searchset <- rownames(searches)

# In Bash, convert to odt with pandoc; e.g.:
# pandoc -o table1.odt table1.tex
print(xtable(searches),
     file = "tables/table1.tex")
```

JMLA Journal of the Medical Library Association
Supplemental content to
J Med Libr Assoc. Jul;107(3):dx.doi.org/10.5195/jmla.2019.622
www.jmla.mlanet.org
© Burns, Shapiro II, Nix, Huber 2019

```
print(xtable(modztable),
    file = "tables/table2.tex")
print(xtable(searchdiffs),
    file = "tables/table3.tex")
```
