# Supplementary material for: Search results outliers among MEDLINE platforms
Source: J Med Libr Assoc. 2019 Jul 1;107(3):364–73. doi: 10.5195/jmla.2019.622 (PMC6579582; doi:10.5195/jmla.2019.622)
Supplement: Appendix D [file jmla-107-364-s004.pdf]

JMLA Journal of the Medical Library Association

Supplemental content to
J Med Libr Assoc. Jul;107(3):dx.doi.org/10.5195/jmla.2019.622
www.jmla.mlanet.org
© Burns, Shapiro II, Nix, Huber 2019

Search results outliers among MEDLINE platforms

Christopher Sean Burns; Robert M. Shapiro II; Tyler Nix; Jeffrey T. Huber

APPENDIX D

Data set formatted for use in the R programming language

```
# These are the search numbers for the 29 sets, October 2018 searches
# The order is PubMed, ProQuest, EBSCOhost, Web of Science, Ovid
s01 <- c(2463875,2475724,2474206,2471152,2460695)
s02 <- c(389955,389674,389410,389120,389537)
s03 <- c(2251033,2238698,2238997,2238118,2232480)
s04 <- c(349598,347192,347205,347183,347195)
s05 <- c(15004,15011,15088,14711,14830)
s06 <- c(41444,40861,40862,40860,40861)
s07 <- c(172,184,184,169,171)
s08 <- c(2838188,2818093,2816030,347183,2815975)
s09 <- c(72297,72641,72987,14711,71594)
s10 <- c(134217,132593,132599,132590,132593)
s11 <- c(2181,2534,2546,2075,2160)
s12 <- c(36503,36497,36475,36475,36483)
s13 <- c(377,377,377,375,377)
s14 <- c(1054,1115,1113,1244,1119)
s15 <- c(35080,35074,35052,35052,35060)
s16 <- c(1423,1423,1423,1423,1423)
s17 <- c(41,41,41,41,41)
s18 <- c(747,678,775,886,745)
s19 <- c(3495,3494,3494,3489,3494)
s20 <- c(111,111,111,111,110)
s21 <- c(16291,18690,16289,16281,16289)
s22 <- c(45782,45748,45716,45678,45741)
s23 <- c(149146,152412,148927,148798,148979)
s24 <- c(600,599,597,0,599)
s25 <- c(12680,12672,12661,12652,12664)
s26 <- c(581,579,577,578,578)
s27 <- c(9432,9421,9406,9397,9411)
s28 <- c(45201,45169,45139,0,45163)
s29 <- c(139714,142991,139521,139401,139568)
```
